# Lack of correlation between parasite burden and key weight metrics in poultry infected with intestinal ascarids

**DOI:** 10.17912/micropub.biology.001197

**Published:** 2024-08-07

**Authors:** JB Collins, Amanda O. Shaver, Etta S. Schaye, Tom Volpe, Leonardo R Nunn, Mostafa Zamanian, Erik C. Andersen

**Affiliations:** 1 Department of Biology, Johns Hopkins University, Baltimore, Maryland, United States; 2 Department of Molecular Biosciences, Northwestern University, Evanston, Illinois, United States; 3 Department of Pathobiological Sciences, University of Wisconsin–Madison, Madison, Wisconsin, United States

## Abstract

*Ascaridia galli* and *Ascaridia dissimilis* are the most common and economically impactful nematode parasites of commercial poultry. These infections rarely cause clinical disease, but reduction in feed conversion efficiency is detected. To determine if feed conversion efficiency reductions correlate with any physiological measures independent of clinical disease, we determined if ascarid infections correlate with changes in the weights of the small intestine, liver, or total animal weight (quantitative measures of animal health). No correlation between parasite burden and these metrics were observed, supporting the concept that feed conversion is the only production metric impacted by ascarid infections.

**Figure 1. Correlation of parasite burdens with small intestine, liver, and total animal weight for chickens and turkeys infected with Ascaridia galli and Ascaridia dissimilis , respectively:**
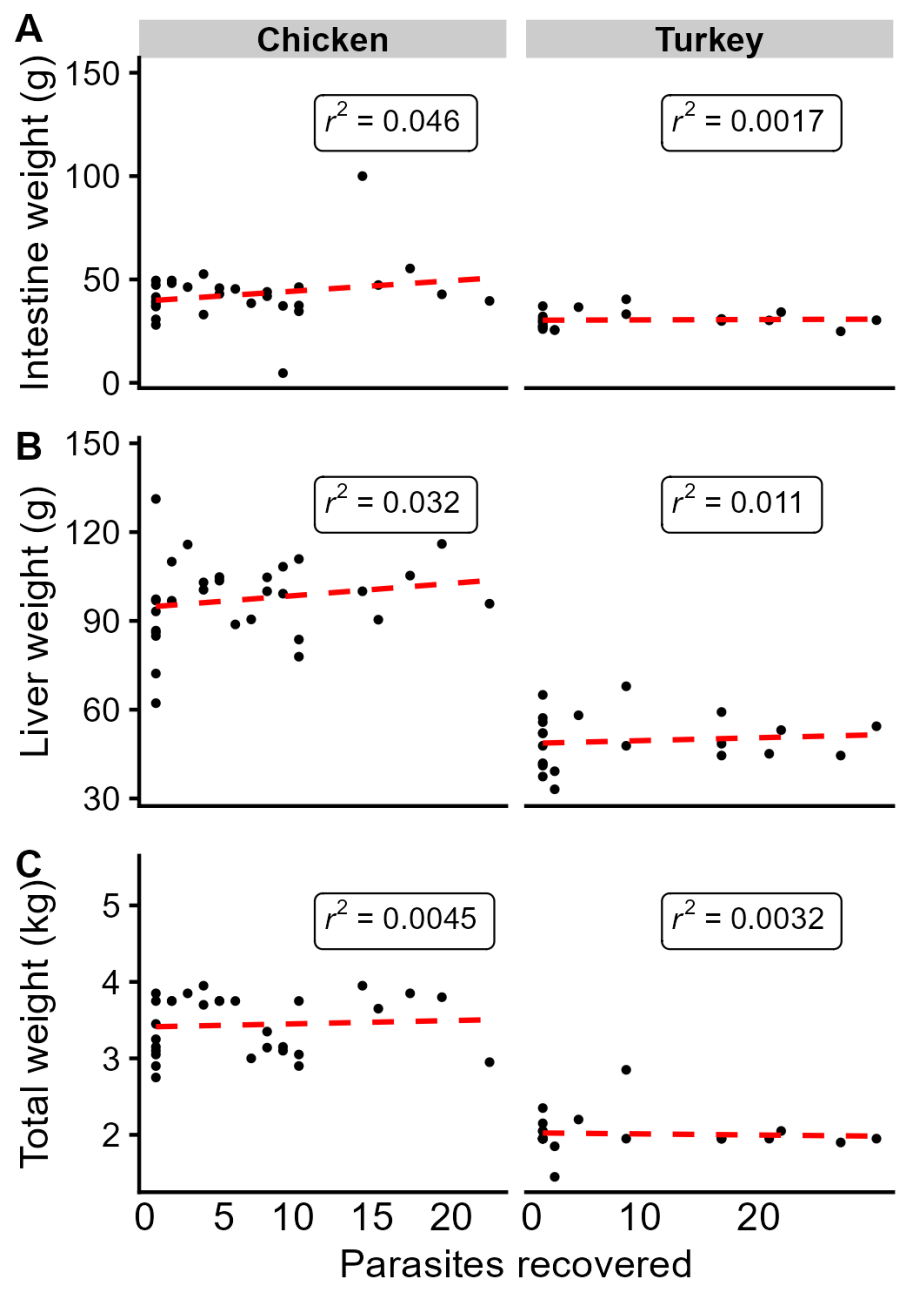
The number of parasites recovered from each bird is plotted on the x-axis, and the weight of the small intestine (A), liver (B), and total weight (C) are shown on the y-axis. A correlation model (dashed line) was applied to each data set, and R ^2^ values of each correlation are shown.

## Description

Studies have indicated that 98.6% of poultry houses are infected with intestinal parasitic nematodes, reaching ubiquity within many houses (Yazwinski et al. 2009; Yazwinski et al. 2013) . *Ascaridia galli* and *Ascaridia dissimilis* , the large ascarids of chickens and turkeys, respectively, are the most economically impactful nematode parasites of poultry. Although most typical infections are often asymptomatic, impacts on feed conversion ratios (FCR), a metric of how efficiently feed is being converted to a unit of production, are often the only indication of infection (Sharma et al. 2018; Collins et al. 2021) . Ascarid infections are acquired from infective embryos in the environment. Embryos hatch in the crop and proventriculus, and larvae are carried to the small intestine where the mucosa is penetrated. Unlike other ascarid species, *Ascaridia* spp. do not undergo obligatory hepato-tracheal migration where young larvae migrate to the liver, followed by the lungs, before being coughed up and swallowed to reach the small intestine to complete development. After multiple molts, young adult larvae emerge into the intestinal lumen where they complete development into reproductive adults and produce embryos, which are then shed in feces. Development of embryos to the infective stage takes three to four weeks, and infective stage larvae can persist in the environment for multiple years. The durability of embryos in the house environment represents the greatest problem to long-term control, because once a house is infected, without a deep clean, infections will continuously cycle through each flock. Therefore, it is necessary to better understand the biology of *Ascaridia* spp., as well as how infections interact with the host, to provide important insights on how infections can be better monitored, informing control strategies.

Despite the lack of overt pathology observed, we asked if ascarid infections have any measurable impacts on weight metrics outside of the known impacts of FCR. Ascarids infect the small intestine, and despite the non-necessity for hepato-tracheal migration, liver foci associated with *Ascaridia* infections have been reported (Norton et al. 1999) . Therefore, we wanted to measure the mass of both the small intestine and liver, as well as total animal weight, to determine if experimental infections impact key weight metrics in a parasite burden-dependent way. A total of 30 chickens and 22 turkeys were infected with *A. galli* and *A. dissimilis,* respectively. Animals were euthanized five weeks after infection at six weeks of age. The host bodies were weighed, followed by the removal of the liver and intestines for subsequent weighing. Parasites were recovered from the intestine and counted. A linear model was fit and correlation values were calculated to determine the relationship between each weight metric and the parasite burden. We found no correlations between parasite burden and the three host weight metrics (small intestine, liver, and total animal weight) in chickens or turkeys. Weak to moderate correlations in chickens and turkeys were observed between small intestine weight and total weight (Chicken r ^2^ =0.25, Turkey r ^2^ =0.54), as well as liver weight with total weight (Chicken r ^2^ =0.41, Turkey r ^2^ =0.56) (Supplementary data). Analysis was performed separately in each host species.

Total bird weight, as well as liver weight, are important metrics associated with marketable parts of poultry. Here, we demonstrate that low to moderate ascarid burdens do not negatively impact weights at the time of slaughter. However, it is important to note that the results presented are not indicative of a lack of any parasite impact. As we have previously demonstrated, subclinical infections with ascarids do not impact host weight but have significant impacts on FCR, greatly increasing the feed volume needed to maintain the same weight as a non-infected host (Collins et al. 2021) . Additionally, we did not examine potential effects on liver lipid reserves or the immune system, which have previously been documented and could be effected by low-burden infections (Sharma et al. 2018) . Correlations made in the current study are drawn from animals initially gavaged with the same number of infective ascarid embryos, so birds with no parasites recovered at necropsy do not represent never-infected animals. Instead, these animals were not infected at the time of necropsy. Future studies should aim to include never-infected animals to compare to infected animals to confirm that parasite burdens do not affect host weight. Overall, we support the concept that FCR decreases are typically the only impact on weight metrics seen in ascarid infections of poultry, but given the precision of the economics of production, the effects of FCR remain a critical concern. Continued efforts to develop new control strategies are needed to mitigate the detrimental effects of ascarids and maintain the economic wellbeing of the poultry industry.

## Methods

***Poultry***

Thirty one-day-old broiler chicks and twenty-two one-day-old white broad-breasted turkey poults were received from Sunnyside Hatchery (Beaver Dam, WI). Animals were allowed to acclimate for one week before infection. A starter/grower ration and water were administered *ad libitum* .

***Parasite infections***

After one week of acclimation, chickens were infected with *A. galli* embryos and turkeys were infected with *A. dissimilis* embryos. Parasite embryos were isolated from commercial chicken and turkey farms, respectively. Each animal was infected with ~200 embryos suspended in 0.5 mL water, delivered into the crop by oral gavage (Collins et al. 2019) .

***Necropsy and worm enumeration***

Five weeks post-infection, animals were humanely euthanized using carbon dioxide asphyxiation, followed by cervical dislocation. Individual animals were weighed and then the body cavity was opened by making lateral cuts along both sides of the breast bone. The small intestine and liver, from the start of the duodenal loop to Meckel’s diverticulum, were carefully removed and weighed separately, after removing intestinal contents. The small intestine was then opened laterally, and all contents were scraped into an individual petri dish. Parasites were carefully collected from intestinal contents, and the total number of parasites was recorded.

***Statistical analysis***

All statistical analyses and correlations were performed in R (4.1.2) (R Core Team 2021) . Linear models were applied using the *lm()* function. All code and data are available at: https://github.com/AndersenLab/2024_PoultryNecropsy_Micropub.

## References

[R1] Collins JB, Jordan B, Baldwin L, Hebron C, Paras K, Vidyashankar AN, Kaplan RM. 2019. Resistance to fenbendazole in Ascaridia dissimilis, an important nematode parasite of turkeys. Poult Sci. 98(11):5412–5415. PMID: 31328783. 10.3382/ps/pez379 31328783

[R2] Collins JB, Jordan B, Vidyashankar AN, Castro PJ, Fowler J, Kaplan RM. 2021. Impact of fenbendazole resistance in Ascaridia dissimilis on the economics of production in turkeys. Poult Sci. 100(11):101435. PMID: 34619579. 10.1016/j.psj.2021.101435 PMC8498455 34619579

[R3] Norton RA, Hoerr FJ, Clark FD, Ricke SC. 1999. Ascarid-associated hepatic foci in turkeys. Avian Dis. 43(1):29–38. PMID: 10216757. 10216757

[R4] R Core Team. 2021. R: A Language and Environment for Statistical Computing [Internet]. https://www.R-project.org/

[R5] Sharma N, Hunt PW, Hine BC, Sharma NK, Chung A, Swick RA, Ruhnke I. 2018. Performance, egg quality, and liver lipid reserves of free-range laying hens naturally infected with Ascaridia galli. Poult Sci. 97(6):1914–1921. PMID: 29562346. 10.3382/ps/pey068 29562346

[R6] Yazwinski TA, Tucker CA, Reynolds J, Johnson Z, Pyle D. 2009. Efficacies of fenbendazole and levamisole in the treatment of commercial turkeys for Ascaridia dissimilis infections. J Appl Poult Res. 18(2):318–324. DOI:https://doi.org/10.3382/japr.2008-00115.

[R7] Yazwinski T, Tucker C, Wray E, Jones L, Johnson Z, Steinlage S, Bridges J. 2013. A survey on the incidence and magnitude of intestinal helminthiasis in broiler breeders originating from the southeastern United States. J Appl Poult Res. 22(4):942–947. DOI: https://doi.org/10.3382/japr.2013-00776.

